# Similarity judgements: the comparison of normative predictions and subjective evaluations – A study of the ratio model of similarity in social context

**DOI:** 10.3389/fpsyg.2024.1335707

**Published:** 2024-05-16

**Authors:** Magdalena Zyta Jabłońska, Andrzej Falkowski, Robert Mackiewicz

**Affiliations:** Faculty of Psychology, SWPS University, Warsaw, Poland

**Keywords:** similarity judgements, ratio model, subjective similarity, objective similarity, positive-negative asymmetry

## Abstract

**Introduction:**

This study examines the consistency between subjective similarity evaluations and the theoretical predictions derived from Tversky’s ratio model of similarity, alongside the impact of additional positive and negative features on perceived similarity to ideal and bad politicians.

**Methods:**

Using a sample of 120 participants, we assessed the similarity of eight candidate profiles to an ideal and bad politician, varying in positive and negative features. Participants’ subjective evaluations were compared with theoretical predictions derived from Tversky’s ratio model. The analysis focused on how candidate and referent valence influenced observed versus theoretical similarity.

**Results:**

Subjective similarity judgments deviated systematically from theoretical predictions, especially for positively featured candidates, indicating a negativity effect. Additional positive features decreased the perceived similarity of favorable candidates to an ideal politician, while additional negative features did not significantly affect similarity judgments of unfavorable candidates.

**Discussion:**

Our findings underscore a significant disparity between subjective and objective similarity judgments, notably for favorable candidates. While the ratio model performs well for unfavorable candidates, its applicability diminishes for favorable ones, emphasizing the role of feature valence in decision-making. Further research on feature valence is crucial for a comprehensive understanding across contexts.

## 1 Introduction

Following William James who stated that “this sense of sameness is the very keel and backbone of our thinking” (James, 1950, p. 459), it can be said that any type of judgement cannot happen without at least some kind of comparison involved. The comparison process is obvious when a task consists in selecting one or more options from an array of alternatives. Still, even if the action does not involve the act of choosing or rejecting, a comparison process is still incorporated in a decision-making and evaluation process (Goldstone, 1999). After all, an evaluation of how good or bad something is requires a certain point of reference to which a given option is compared.

As the judgements of similarity are so fundamental for evaluation and decision-making (e.g., they are also an intrinsic part of plausibility judgements, where an imagined event is compared to the actual world and based on that its probability is estimated), it is especially important to analyse various aspects of similarity estimates. In our article, we draw on Tversky’s ratio model of similarity (Tversky, 1977) and compare the normative predictions of the model (i.e. objective similarity) with actual estimations of similarity made by study participants (subjective similarity). Furthermore, joining together similarity research with the literature on positive-negative asymmetry, we analyse how additional positive and negative features added to descriptions of differently valenced profiles affect their similarity to an ideal object and a bad object.

### 1.1 Tversky’s features of similarity model and its theoretical predictions

The model proposed by Tversky (1977) assumes that objects can be defined as sets of features. These features can vary in nature, including nominal (e.g., eye colour), binary (e.g., voiced and voiceless consonants), and graded (representing different levels of a person’s honesty). The similarity between two such sets is a function of features that are common in both and those that belong only to one of the sets. The similarity between objects *a* and *b* can be defined as matching function described in Equation 1 (Tversky, 1977, p. 330):


(1) s⁢(a,b)=f⁢(A∩B,A-B,B-A)

The similarity *s* between *a* and *b* is a function *f* that matches the features belonging to both *a* and *b* (*A*∩*B*), the features belonging only to *a* (*A* − *B*) and the features belonging only to *b* (*B* − *A*). Function *f* translates these sets into a similarity scale. Theoretically, any function is possible as long as it preserves the monotonicity axiom: adding common features and/or deleting distinct features should increase similarity between the sets. One such function proposed by Tversky (1977) is the ratio model, which calculates the similarity as the ratio of the number of common features and the number of all features belonging to both objects. The ratio model can be described as:


(2) $$s\,\left(a,b\right)=\frac{f\,\left(A\cap B\right)}{f\,\left(A\cap B\right)+\alpha \,f\,\left(A-B\right)+\beta \,f\,\left(B-A\right)}$$

In this equation *s*(*a,b*) refers to some similarity scale that expresses the similarity with numbers. The parameters refer to the salience of different features (*f*) and the importance of common and distinctive features (represented as weights: α for features belonging to *a*, and β for features belonging to *b*).

The general formula presented in Equation 2 can be transformed into many different specific models and their actual form depends on the parameters. For example when α = β = 1, then *s*(*a,b*) is equal to *f*(*A*∩*B*)/*f*(*A*∪*B*) (see Tversky, 1977, p. 333). In the simplest possible case, *f* can be treated as a count function that returns numbers of features. In such a case similarity would equal just the ratio of common features of both objects and the features that belong to both:


(3) $$s\,\left(a,b\right)=\frac{n\,\left(A\cap B\right)}{n\,\left(A\cup B\right)}$$

We may call Equation 3 the ratio model and it is the simplest theoretical model of similarity as it takes into account only the number of features.

### 1.2 Positive-negative asymmetry in similarity judgements

Tversky’s model addresses the disparity between *s*(*a,b*) and *s*(*b,a*) by introducing the concept of focusing. When comparing *a* to *b*, *a* serves as the subject and *b* as the referent, resulting in the subject’s features being weighted more heavily than those of the referent. However, the model does not consider the valence of objects being assessed for similarity, in other words similarity is defined as the function of common and distinctive features regardless of the fact that they may be positive or negative.

Numerous studies in social and cognitive psychology show that negative features exert a more potent influence and have a stronger impact on judgement than positive ones (Baumeister et al., 2001). For instance, negative emotions elicit greater intensity than positive ones, leading to heightened attention and memory allocation towards negative stimuli (Rozin and Royzman, 2001). Negative information also necessitates more cognitive processing, resulting in deeper encoding and stronger memory formation (Vaish et al., 2008). In the realm of decision-making, loss aversion, a key concept in prospect theory (Kahneman and Tversky, 1979), suggests that individuals tend to strongly prefer avoiding losses over acquiring gains. This often leads to more pronounced avoidance behaviours following the exposure to a negative stimulus, in contrast to approach tendencies associated with a willingness to engage after contact with a positive stimulus or situation (Scerrati et al., 2023). Explanations for the negativity effect include evolutionary survival mechanisms and the higher informativeness and diagnosticity of negative information (Fiske, 1980; Skowronski and Carlston, 1989; Taylor, 1991).

Despite the prevalence of negative information, certain contexts exhibit a positivity bias. Studies indicate that individuals tend to recall and perceive positive events more frequently than negative ones (Carstensen and Mikels, 2005), maintaining a generally positive outlook on others and the world (Hoorens, 2014). The research on positivity and negativity effects converges in studies on positive-negative asymmetry, investigating factors influencing the impact of negative or positive information (Peeters, 1971; Peeters and Czapinski, 1990; Blanz et al., 1995; Mummendey et al., 2000).

Expectancy contrast theories and the figure-ground hypothesis provide insights into which features – positive or negative – predominate (Lau, 1982, 1985; Skowronski and Carlston, 1989). According to these theories, less common features stand out more and are more noticeable. As people generally expect other people and the world itself to be rather good than bad (the normative positivity bias), the negative traits or events – if they happen – stand out more and are more diagnostic than their positive counterparts (the negativity effect). However, in contexts where negativity prevails, small positive occurrences can still have a significant impact (Kellermann, 1984).

Psychophysical principles such as the ratio-difference principle (Gescheider, 1997) and the principle of diminishing sensitivity (Tversky and Kahneman, 1992) underpin these effects. They suggest that individuals demonstrate reduced sensitivity to differences in probabilities or magnitudes as the values involved increase. For instance, the act of turning on a small lamp in a dark room can have a significant impact. However, when the light level increases by the same degree in a well-lit room, it may go unnoticed. Notably, these principles are also consistent with Tversky’s ratio model of similarity, which predicts changes in similarity based on the ratio of common and distinctive features.

### 1.3 The comparison of objective and subjective similarity estimates and the rationale for the study

Falkowski and Jabłońska (2018) and Falkowski et al. (2021) were among a few researchers who adopted Tversky’s feature-based model of similarity in the context of positive-negative asymmetry. Their investigations delved into how adding or removing positive and negative information influenced the evaluation of fictitious political candidates (Falkowski and Jabłońska, 2018) and cities (Falkowski et al., 2021), depending on the initial favourability of objects under analysis. The results demonstrated that, for objects initially perceived as favourable, negative information had a more substantial impact, whereas for those initially regarded as unfavourable, positive features induced more pronounced changes in evaluation. Importantly, however, the researchers did not compare normative predictions of the ratio model with actual judgments, that is perceived similarity. Therefore, the current study aims to fill this research gap by examining the alignment between the model’s normative predictions (objective similarity) and empirical evidence (subjective similarity).

Our point of departure is the theoretical model presented in Equation 3. According to the model, if people base their judgments of similarity solely on the number of features, the subjective assessments of similarity should be consistent regardless of whether the object and its referent are positive or negative. Consider evaluations of two political candidates. Candidate A possesses two positive attributes and seven negative attributes, suggesting a predominantly negative evaluation. Conversely, Candidate B exhibits two negative features and seven positive features, indicating a predominantly positive evaluation. According to the ratio model, when comparing each candidate to the best and worst possible candidates, the predicted similarity remains the same. For Candidate A, who shares two attributes with the best possible candidate (assumed to possess all positive attributes), the similarity is calculated as 2 / 9 = 0.222. Similarly, Candidate B shares two features with the worst possible candidate (presumed to have all negative features), resulting in the same similarity of 0.222. However, the model overlooks the valence of the candidates. Assessing similarity between Candidate A and the best possible candidate necessitates focusing on positive attributes, while evaluating Candidate B against the worst possible candidate requires focusing on negative attributes.

The studies on the negativity effect show that negative features have a higher impact on judgements than positive ones. Consequently, assessments of similarity between actual candidates and their best or worst versions should reflect the valence of features. In the study described below, we examined how participants assessed the similarity of fictitious politicians to the best and worst possible candidates, assuming these extreme candidates possess solely positive or negative features. Previous studies indicate that individuals tend to avoid extreme judgments (Christenfeld, 1995; Shaw et al., 2000), suggesting that evaluations of political candidates should diverge from predictions based solely on the number of features.

The relationship between predictions based on the numbers of features and subjective judgments does not have to be linear. For example, it may be expressed by power function in the same way as in the prospect theory (Tversky and Kahneman, 1992). Such a parameter could reflect the diminished sensitivity to added common and distinctive features as presented in Equation 2. However, determining the specific value of this parameter is an empirical matter and multiple solutions are plausible. One potential scenario is that sensitivity parameters vary between object *a* and object *b*, resulting in α ≠ β. The other possibility is that parameters α and β do not operate upon objects but upon the types of features. Another possibility is that α = β but there is some additional parameter scaling the valuation of some features. Regardless of the way the parameters are incorporated into the model, we hypothesise that negative features will exert a greater influence on valuations than positive features. Therefore, we predict that subjective assessments of similarity should generally be lower than theoretical predictions due to diminishing sensitivity to a bigger number of features, although this effect should be mitigated by the negativity effect for unfavourable candidates. Hence, we propose Hypothesis 1:

**H1:** The subjective similarity between an object and its referent should systematically deviate from theoretical predictions in that the evaluations of favourable candidates deviate higher from theoretical predictions than the evaluations of unfavourable candidates.

The predominance of negative features should be also visible in the differences between evaluations of objects with additional positive and negative features. If negative features hold greater value, then additional negative features should exert a stronger influence on evaluations compared to additional positive features. Accounting for diminishing sensitivity, the effect should be more pronounced for favourable candidates. In other words, we expect that a change from two to four negative features in case of favourable candidates (with seven or nine positive features) will be more potent than a change from two to four positive features in case of unfavourable candidates (with seven or nine negative features). We formulate this prediction as Hypothesis 2:

**H2:** Additional negative features will lead to greater changes in subjective similarity judgements compared to the effect of additional positive features. The effect will be especially visible for objects that have more positive than negative features.

In order to test these predictions, we need to make certain assumptions. First, an object (characterised by various positive and negative features) will always be a target of comparison and it will be compared to an ideal or bad referent. Such an assumption is necessary to account for the focusing hypothesis which predicts that the direction of comparison affects similarity judgements. Second, we assume that all features characterising an ideal object are positive and that all features characterising a bad object are negative. Third, the valence of the referent (ideal or bad) will determine the valence of common and distinctive features that an object of comparison has with its referent. For instance, for comparisons to an ideal object (referent), an object with two positive and seven negative features will have two common features and seven distinctive ones. If the same object is compared to a bad referent, then it shares seven common features but is different by two distinctive features, not present in the set of the referent.

We selected political candidates as objects in our study. There are a few reasons for such a decision. First, the evaluation of political candidates is a process that most people at least occasionally undertake and which has important consequences on the functioning of states and the lives of others. Second, people are used to analysing and comparing political candidates with regard to various criteria and features. Finally, people are quite unanimous with regard to the most typical features characterising “an ideal politician” and “a bad politician” across different cultures (Miller et al., 1986; Cwalina et al., 2005), using similar competence- and morality-related traits to describe them (Skowronski and Carlston, 1987, 1989; Wojciszke, 2005; Fiske et al., 2007).

## 2 Materials and methods

### 2.1 Participants

Our *a priori* power analysis with the assumption of a medium effect size, an α error probability of 0.05, and a power of 0.95 indicated a required sample size of 76 participants to test Hypothesis 1 and a sample of 107 participants to test Hypothesis 2 (Faul et al., 2007). In total, 120 participants (57% female), aged 18–54 (*M* = 24.41, SD = 5.45), took part in the experiment. Participants were recruited from university students and were not remunerated for taking part in the study. The research was conducted in compliance with APA ethical guidelines (American Psychological Association [APA], 2010) and informed consent was obtained from all participants. The Ethical Board of the Department of Psychology approved the study. On average, participants were moderately interested in politics (*M* = 4.33, SD = 2.79, on a 11-point Likert scale) and were neither extremely left- or right-wing oriented (*M* = 4.42, SD = 1.94, on a 11-point Likert scale).

### 2.2 Procedure

The participants were assessed in small groups of 20–30 individuals in a classroom setting. Each participant received a booklet containing instructions, a consent form, and the experimental materials. After the participants had familiarised themselves with the instructions they were informed about the nature of the task. In the instructions they could read that the aim of the study was to investigate how people evaluate different politicians and their task would be to express what they think about two fictitious candidates. The participants filled in their answers in the provided booklets and the whole study lasted about 15 min.

### 2.3 Materials and design

Eight short, narrative descriptions of political candidates were used (see Supplementary Appendix 1A). Candidates differed in the proportion of positive and negative features that described them. Table 1 presents candidate profiles used in the study. Columns 1 and 2 show the number of features characterising each candidate, whereas columns 3 and 4 present the values of similarity to an ideal and bad politician calculated with the ratio model in Equation 3.

**TABLE 1:** Similarity to the positive and negative referent of candidate profiles used in the study.

Number of positive features	Number of negative features	Similarity to an ideal politician	Similarity to a bad politician
2	9	0.18	0.82
2	7	0.22	0.78
4	9	0.31	0.69
4	7	0.36	0.64
7	4	0.64	0.36
9	4	0.69	0.31
7	2	0.78	0.22
9	2	0.82	0.18

Similarity is calculated based on the ratio model of similarity (*S* = *x*(*x* + *y*)). The candidates are organised from the highest to the lowest similarity to an ideal politician (column 3).

A candidate characterised by seven positive and two negative features (7+2− for brevity) served as the baseline candidate for all other favourable candidates (i.e., those with more positive than negative features). Consequently, all other favourable candidates shared the same features as Candidate 7+2− but with additional two positive or negative features (resulting in candidate profiles featuring either seven or nine positive features and two or four negative features). Likewise, Candidate 2+7− served as the baseline profile for other unfavourable candidates (who possessed either two or four positive features and seven or nine negative features). Thanks to such a design we could later analyse how additional positive and negative features affect similarity judgements (Hypothesis 2).

The features used to create candidate profiles were based on previous research in which we determined the inner structure of the concepts of “an ideal politician” and “a bad politician” (Falkowski and Jabłońska, 2018). Furthermore, particular sets of features were tested in a pilot study in order to make sure that the positive and negative features used to create candidate profiles differed in their valence but not their diagnosticity. The features used in the study as well as the results of a pilot study are presented in Supplementary Appendix 1B.

Each participant evaluated two candidates, one with the majority of negative and one with the majority of positive features. The candidates evaluated by the participant were chosen in such a way that the theoretical similarity of a positive candidate to the best possible one equalled the theoretical similarity of the negative candidate to the worst possible one. Therefore, the same participant assessed the candidate with two positive and seven negative traits and the candidate with two negative and seven positive traits. In result the valence of the evaluated candidate was a within – group independent variable. Each participant evaluated the candidate’s similarity twice – how similar the candidate was to the worst and to the best possible candidate. So the object of comparison served as the second within subject variable. The number of positive traits and the number of negative traits were independent between-group variables.

At the beginning of the study, participants filled in demographic information (age and gender) as well as answered questions on their political engagement (*On a scale from 0 to 10, how interested in politics are you?* with 0 *very disinterested* and 10 *very interested*) and political beliefs (*On a scale from 0 very left-wing to 10 very right-wing, how would you describe your political beliefs?*). Later, participants were asked to carefully read a candidate profile and evaluate it with regard to the candidate’s similarity to an ideal politician and a bad politician. Here, we asked participants to reflect on the features typical for an ideal (bad) candidate and decide how similar a given profile was to this ideal (bad) representation [*On the scale from 0 to 10, how similar is the candidate to an ideal (bad) politician?* with 0 *very dissimilar* and 10 *very similar*].

## 3 Results

### 3.1 Subjective vs. objective similarity measures

**FIGURE 1:**
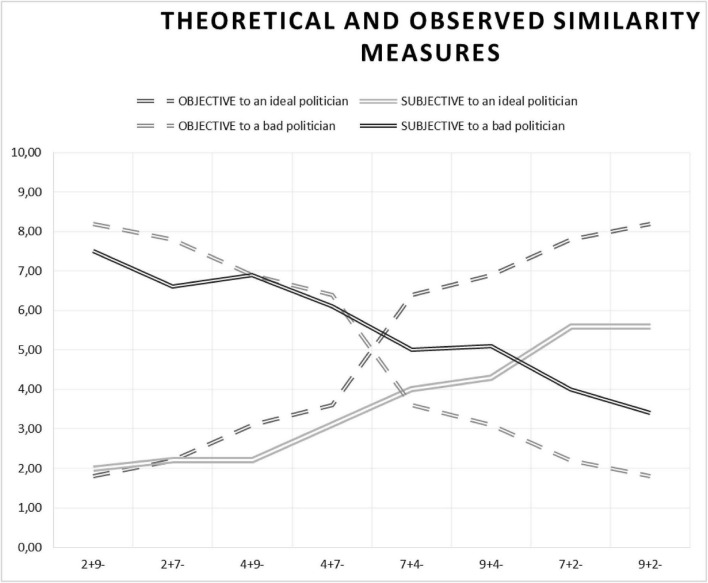
The figure illustrates the objective (theoretical) and subjective (observed) similarity to both an ideal and bad politician for candidate profiles differing in the number of positive and negative features attributed to them (*x*-axis). Dashed lines depict the theoretical predictions, while solid lines represent empirical findings. Lighter lines indicate similarity to an ideal politician, whereas darker lines signify similarity to a bad politician.

Our first objective was to assess the consistency between participants’ subjective evaluations of similarity and the model-based predictions of the ratio model of similarity. To prepare for data analysis, we made adjustments to our variables. First, we rescaled the theoretical similarity measures from a range of 0 to 1 to match the scale of 0 to 10, aligning them with the subjective similarity measures. Both predicted and observed similarity measures for similarity to an ideal and bad politician are presented in Table 2. Additionally, Figure 1 offers a graphical depiction of both the normative and observed similarity judgements for candidate profiles varying in positive and negative features.

**TABLE 2:** The objective (theoretical) and subjective (observed) similarities to an ideal and bad politician.

*N*	Candidate profile		Similarity to an ideal politician			Similarity to a bad politician		
	Positive features	Negative features	Objective similarity	Subjective similarity		Objective similarity	Subjective similarity	
				*M*	SD		*M*	SD
30	2	9	1.82	2.03	2.042	8.18	7.53	2.255
30	2	7	2.22	2.20	1.583	7.78	6.60	2.441
30	4	9	3.08	2.17	1.733	6.92	6.93	2.273
30	4	7	3.64	3.07	2.658	6.36	6.07	2.516
30	7	4	6.36	3.93	2.119	3.64	5.00	2.236
30	9	4	6.92	4.21	2.483	3.08	4.93	2.498
30	7	2	7.78	5.47	2.063	2.22	4.07	2.163
30	9	2	8.18	5.73	2.016	1.82	3.53	1.943

**FIGURE 2:**
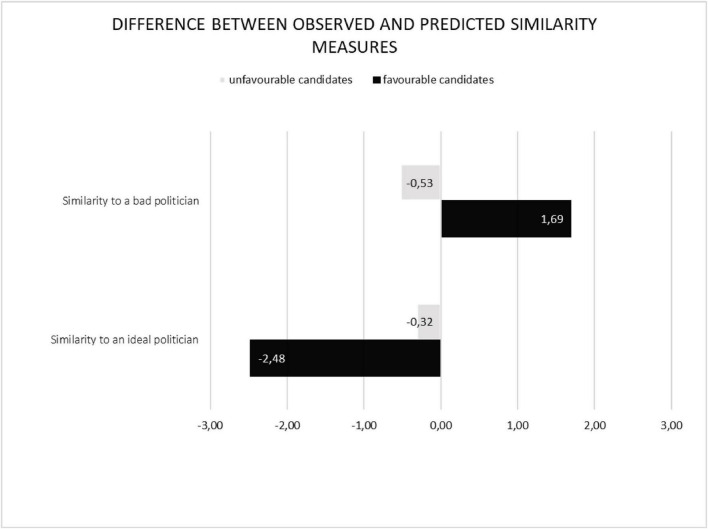
The differences between observed and predicted similarity measures for similarity to an ideal and bad politician for favourable and unfavourable candidates. Positive values indicate that the observed similarity values are higher than the predicted values.

Subsequently, we computed new variables aimed at capturing the disparities between observed and theoretical similarity scores concerning evaluations of similarity to an ideal and a bad politician. We subtracted theoretical similarity measures from observed ones. Hence, positive values would indicate that a subjective similarity measure is higher than the theoretical prediction, while negative measures would show that someone rated the similarity as smaller than predicted by the ratio model. The differences between observed and predicted similarity measures for both favourable and unfavourable candidates are illustrated in Figure 2.

In the next step, we conducted a repeated-measures ANOVA, with candidate valence (favourable/unfavourable) and referent valence (comparison to an ideal/bad politician) as within-subject factors, and with the difference between the theoretical and observed similarity measures as the dependent variable.

The rmANOVA analysis revealed a significant effect of referent valence, *F*(1,118) = 53.665, *p* < 0.001, η^2^ = 0.313, and a significant interaction between candidate valence and referent valence, *F*(1,118) = 85.305, *p* < 0.001, η^2^ = 0.420. However, the effect of candidate valence was found to be non-significant, *F*(1,118) = 0.116, *p* = 0.734, η^2^ = 0.001. Further examination of the main effect of referent valence indicated greater disparities between predicted and observed similarity measures for comparisons to an ideal politician (*M* = −1.395; SD = 2.128) than to a bad politician (*M* = 0.580; SD = 2.303). Notably, candidates’ similarity to an ideal referent was significantly underestimated [with *t*-test values for one sample: *t*(238) = −9.100, *p* < 0.001, Cohen’s *d* = −0.589], while their similarity to a bad politician was significantly overestimated [*t*(239) = 0.3.569, *p* < 0.001, Cohen’s *d* = 0.230].

Following this, a *post hoc* analysis was conducted on the identified interaction between candidate valence and referent valence. The findings revealed a significant discrepancy between predicted and actual values when comparing favourable and unfavourable candidates to an ideal politician. Specifically, favourable candidates (*M* = −2.478; SD = 2.168) exhibited a much larger difference than unfavourable candidates (*M* = −0.528; SD = 2.387), *F*(1,118) = 83.610, *p* < 0.001, η^2^ = 0.415. This suggests that the evaluations of favourable candidates were more negative than the anticipated, with lower similarity to an ideal politician. Similarly, in the context of similarity to a bad politician, favourable candidates (*M* = 1.693; SD = 2.209) displayed greater differences from predicted values compared to unfavourable candidates (*M* = −0.533; SD = 2.396), *F*(1,118) = 64.883, *p* < 0.001, η^2^ = 0.355, with greater than expected similarity to a bad politician.

The analyses provided support for Hypothesis 1, which anticipated systematic variations between the perceived and theoretical similarity measures, with the differences being more pronounced for favourable candidates compared to unfavourable ones. As demonstrated in the study, positive candidates were generally perceived as less similar to an ideal politician and more similar to a bad politician compared to what the ratio model would predict. Furthermore, participants consistently undervalued favourable candidates, perceiving them as more akin to a bad politician and less akin to an ideal politician. Conversely, evaluations of unfavourable candidates aligned closely with predicted values for similarity to an ideal politician, yet their similarity to a bad politician was underestimated, resulting in a more positive perception than warranted. For instance, if we analyse the best (9+2−) and the worst (2+9−) candidates from the set, we can observe that while the worst candidate received a fair evaluation, the best one was deemed only “mediocre,” with subjective similarity to an ideal politician of *M* = 5.6. Interestingly, this rating was even lower than the normative prediction for a candidate with two positive features less and two negative features more (7+4−), whose predicted similarity equalled 6.4. This observation suggests that people generally hold higher standards for candidates perceived as “good.”

Furthermore, the results indicate that negative features hold more significance than positive ones. Specifically, when an object is positive, its subjective similarity to a positive referent decreases by 2.478 points on the scale, while its similarity to a negative referent increases by 1.693 points. These differences are notably larger than those observed for predominantly negative candidates: *M* = −0.528 for comparison with a positive referent and *M* = −0.533 for comparison with a negative referent. Consequently, candidates with predominantly negative features align with the predictions of the ratio model. Conversely, candidates with predominantly positive features are assessed more negatively than anticipated. In summary, negative features exert a stronger influence, accurately reflecting the negativity of unfavourable candidates while amplifying the negativity of favourable candidates.

### 3.2 The effect of additional positive and negative features on subjective similarity judgements

The second aim of the study was to test the effect of additional positive and negative features on perceived similarity to an ideal and bad politician. According to the normative predictions of the model, feature valence is important insofar as it determines whether a particular feature belongs to the set of common or distinctive features. Otherwise, the model implies a symmetrical effect of positively and negatively valenced features. However, as shown in the previous section, there were visible differences in the way favourable and unfavourable candidates were evaluated. Thus, we wanted to investigate how additional positive and negative information about a candidate influences their similarity to an ideal and bad politician, depending on their initial valence.

In order to do that, we conducted a mixed design ANOVA to examine the interaction effect of the number of features (four levels), candidate valence (favourable/unfavourable), and referent (ideal/bad politician) on similarity judgements. The first factor constituted a between-subjects variable, while all others were within-subjects variables. The results revealed a significant interaction effect, *F*(3,115) = 6.763, *p* < 0.001, partial η^2^ = 0.150. Consequently, we performed *post-hoc* tests (utilising the Sidak adjustment) to test Hypothesis 2 and examine how changes in the number of positive and negative features impact similarity to an ideal and bad politician for favourable and unfavourable candidate profiles. The means and standard deviations are presented in Table 2.

Overall, there were no differences between particular profiles in case of unfavourable candidates in their similarity to an ideal politician, *F*(3,115) = 1.569, *p* = 0.201, partial η^2^ = 0.039, or a bad politician, *F*(3,115) = 1.987, *p* = 0.120, partial η^2^ = 0.049. Conversely, for favourable candidates, noteworthy differences emerged among specific profiles concerning their similarity to an ideal politician, *F*(3,115) = 4.965, *p* = 0.003, partial η^2^ = 0.115. Specifically, significant differences were evident between candidate profiles 7+2− and 7+4− (*p* = 0.047), as well as 9+2− and 7+4− (*p* = 0.012). Although the disparity between candidates 9+2− and 9+4− did not reach statistical significance (*p* = 0.052), it followed a similar trend, demonstrating a more pronounced effect of additional negative features. Regarding the similarity to a bad politician for favourable candidates, while the overall effect of the number of features was significant, *F*(3,115) = 3.006, *p* = 0.033, partial η^2^ = 0.073, subsequent post-hoc tests did not reveal any significant differences.

## 4 Discussion

### 4.1 The comparison of objective and subjective similarity estimates

Our research had two main objectives. First, we wanted to test the predictions of the ratio model of similarity developed by Tversky (1977) with regard to positively- and negatively valenced objects. Although highly influential, Tversky’s feature-based approach to similarity was rarely adopted to the studies on positive-negative asymmetry. We identified this as an interesting research gap and designed a study in which we compared the predicted similarity estimates, calculated based on the ratio model, with actual similarity judgements made by study participants. We hypothesised that subjective estimates would consistently deviate from theoretical values, with these deviations being more pronounced for favourable candidates. To test this hypothesis, we created various candidate profiles differing in the number of positive and negative features they exhibited. Study participants were then asked to evaluate how similar a given candidate was to both an ideal politician and a bad politician, which served as referents. Objective similarity judgments were calculated using Equation 3, which defines similarity as the ratio of common features to all features. For instance, a candidate with two positive and seven negative features would be assigned a similarity of *S* = 0.22 to an ideal politician and *S* = 0.78 to a bad politician. Subsequently, we compared these normative predictions with the subjective similarity judgments provided by participants.

The similarity with both the ideal and bad referent of candidates with a predominance of negative features did not differ from theoretical predictions. However, we observed systematic deviations between subjective estimates and theoretical predictions for candidates with a predominance of positive features. These candidates were evaluated as more negative, as indicated by arithmetic calculations: more similar to the negative referent and less similar to the positive referent. This result clearly demonstrates the negativity effect and supports our Hypothesis 1. However, this result seems challenging to reconcile with the ratio-difference principle. If participants’ responsiveness to additional features diminishes as the number of features increases, then the relationship between theoretical and subjective estimates of similarity should remain consistent regardless of the valence of the referent.

To illustrate this point, let’s examine the relation between subjective and objective similarity in comparisons with the ideal candidate as depicted in Figure 1. The least favourable candidate, judged by participants, was characterised by nine negative and two positive features. When participants assessed the similarity between this candidate and the ideal, they based their evaluations on the proportion of positive features relative to the total number of features. As the number of positive features increased, so did the subjective estimates of similarity to the ideal politician. However, the slope of the subjective estimates was observed to be lower than that of the objective estimates. We refrain from providing precise estimates of this difference between slopes due to the limitation of having only one dataset. Nevertheless, we could illustrate this tendency with a linear function (although theoretically, the relation may not be strictly linear): *W*(f+) = *k*(f), where *W* denotes the weight assigned to positive features (f+), and *k* represents the value of this weight. Regardless of the specific value of *k*, our data consistently indicate that *k* < 1. Consequently, the subjective similarity between the candidate with nine positive and two negative features appears to be lower than the proportion 2/11.

However, the ratio-difference principle does not seem to hold for the comparisons to the worst possible (bad) candidate. Consider the candidate 9−2+ and the estimate of their similarity to the bad referent – the empirical result does not differ from theoretical prediction: 9/11. If participants applied the ratio-difference principle for comparisons to the bad referent, then the reduction of negative features should result in increased sensitivity (fewer features should be more visible). Consequently, favourable candidates (such as 9+2−) should be even more similar to the bad referent than negative ones. But this was not the case. If we used a linear estimate of the same kind as we proposed for positive features, then the value of *W*(f−) should be *k* = 2. The value of *k* = 2 is only a rough estimate but it can still be treated as the estimate of negativity effect in our study. We may treat the value *k* = 2 as the estimate of the negativity effect, but this poses some problems with the ratio-difference principle. If the effect of negative features is strong, then the negative candidates should be evaluated as more negative than predicted theoretically. Our data do not show this. One reason could be that the participants were assessing the similarity on the scale from 0 to 10. It is possible that they were avoiding the endpoints of the scale, so their estimations did not exceed theoretical predictions. Considering that the descriptions we used were highly negative, there was no room for exceeding the predictions from the ratio model. Nonetheless, this is evidently a challenge that should be addressed in further research.

The negativity effect that we observed in evaluations of positive candidates follows Epstein’s research on cognitive-experiential self-theory (Epstein, 1990; Kirkpatrick and Epstein, 1992) and his distinction into the experiential and rational system of information-processing. While the latter is based on the rules of logic and evidence, the former uses simple heuristics and emotions. Both systems operate also using different criteria for decision-making, with the experiential model using concrete exemplars gathered in the process of emotion-laden experiential learning, and the rational system using abstract symbols. It seems likely that whereas the judgements of unfavourable candidates relied on the number of features that characterised them, the judgements of favourable candidates activated stronger the representations of an ideal politician compared to which evaluated entities were perceived as less attractive than they might seem.

Importantly, cognitive-experiential theory was also used to explain paradoxical effects in probability judgements, extending norm theory (Kahneman and Miller, 1986), also with regard to positive-negative asymmetry as in the case of unexpected changes in probability judgements about chances of drawing winning or losing tickets. As shown in studies conducted by Kirkpatrick and Epstein (1992), cognitive-experiential self-theory was more accurate in probability judgements than norm theory, explaining effects not anticipated by the latter. Importantly, this higher predictive power relied on subjective probability estimates, not included in norm theory. Although CEST predictions did not fully mirror our findings with regard to judgements of differently valenced events – showing effects for both positive and negative events – it stresses the role of subjective probability estimates in probability judgements which diverge from the expected, normative predictions.

Finally, although probability judgements and similarity judgements have rarely been studied together, the former cannot take place without the latter. This problem was addressed by Galesic et al. (2018) who using descriptions of fictive citizens of 15th century Florence showed that similarity between the profiles as well as the likelihood of them belonging to the same family coincide. Furthermore, as shown by recent studies on counterfactual plausibility, the perceived likelihood of an event to happen is predicted by the perceived similarity between the possible world in which the imagined situation is to occur and the actual world (De Brigard et al., 2021). Importantly, the shifts in plausibility estimates (i.e., the same event is perceived as more likely or unlikely to happen) can be explained with regard to the shifts in attention that people direct toward common and distinctive features between the imagined counterfactual event and the actual reality. As such they fit well Tversky’s contrast model of similarity and the ratio model (Tversky, 1977).

Going back to our findings, it is worth mentioning that while the evaluations of unfavourable candidates adhered closely to the theoretical model and were relatively fair, albeit less varied, assessments of favourable candidates were notably harsh. Even the most favourable candidate from the set (9+2−) was perceived as merely average, hovering around the midpoint. This suggests that while people are rather objective and unanimous in their estimations of what is “bad,” they are less willing to label something as “good.” This asymmetry can be explained with regard to the distinction into sufficient and necessary conditions for the occurrence of an event, which taps directly into the previously discussed research on plausibility judgements. A necessary condition is one that must be present for the event to happen, although it does not guarantee the event’s occurrence, while a sufficient condition is one that will inevitably produce the event. For positive objects, the set of desirable traits (i.e., necessary conditions) is potentially limitless, as hypothetically an object cannot possess “too many” good qualities. However, it seems unlikely that individuals base their judgments solely on sufficient conditions when assessing similarity (although individual differences may exist, as noted by Schwartz et al. (2002). The situation looks, however, different when one thinks about features of a negative object (in our case a bad politician). Here, the list of necessary conditions not to choose an option is rather short. Moreover, the likelihood of a sufficient condition also appears to be higher. Thus, although individuals may fall into the categories of maximisers and satisficers, with the latter being more content with their decisions (Schwartz et al., 2002; Iyengar et al., 2006), it appears that when evaluating positive objects, a satisfying strategy is not our natural modus operandi (as evident in Figure 1). Future research should delve into these issues, exploring the interplay between similarity and probability judgments of objects with different valences, as well as potential disparities between maximisers and satisficers in their similarity and plausibility judgments.

### 4.2 The effect of additional positive and negative features on similarity judgements

Our second objective was to delve deeper into how the inclusion of additional positive and negative features influences the perceived similarity to an ideal and bad politician. The ratio model of similarity is valence-insensitive as it predicts a symmetrical effect of positive and negative features on similarity as long as they have the same proportion of common and distinctive features. In other words, according to normative predictions two additional positive features added to the object characterised by two positive and seven negative features should have the same effect as two negative features added to the object characterised by two negative and seven positive features.

However, as suggested by a substantial body of research on positive-negative asymmetry, good and bad are rarely symmetrical. First – according to expectancy-contrast theories and figure-ground hypothesis (Lau, 1982, 1985; Skowronski and Carlston, 1989), a less frequent feature will stand out more and exert a stronger effect compared to its more frequent counterpart. Second, all else being equal, negative features should elicit greater changes in similarity judgments than their positive counterparts. Thus, we hypothesised that while additional positive features should impact the perception of negative objects, and additional negative features should affect the perception of positive objects, only the latter effect would be significant.

To test these predictions, we analysed how the changes in the number of positive and negative features describing favourable and unfavourable candidates influenced similarity judgements. Additionally, we ensured the diagnosticity of the additional features was controlled, ensuring any potentially asymmetrical effect stemmed from feature valence and not their diagnosticity. The findings supported Hypothesis 2, demonstrating that while changes in the number of negative features (e.g., a change from two to four features) decreased the perceived similarity of favourable candidates to the image of an ideal politician, parallel changes in the number of positive (and negative, for that matter) features did not meaningfully impact similarity judgments about unfavourable candidates.

The study points to two instances of positive-negative asymmetry. The fact that negative features had an impact on the perception of favourable candidates and not the unfavourable ones can be explained with the previously mentioned ratio-difference principle (Quattrone and Tversky, 1988) and the predictions of the ratio model of similarity. A change from two to four negative features characterising a favourable candidate produces a ratio of change equal to 2, whereas the same two negative features added to the portfolio of an unfavourable candidate (e.g., a change from seven to nine negative features) leads to a ratio of change equal to 1.3. Importantly, a similar pattern of changes within the smaller and the bigger set was not observed for additional positive features.

The lack of the effect of additional positive features on similarity judgements about bad objects is the second instance of positive-negative asymmetry observed in the study. Importantly, the effect cannot be explained with higher diagnosticity of negative features (Skowronski and Carlston, 1987; Wentura et al., 2000; Rozin and Royzman, 2001), as this was carefully controlled for. However, it is possible that although the two positive and two negative features had comparable diagnosticity in isolation, their impact changed due to the context in which they appeared (i.e., when added to other positive and negative features). Such an assumption is in line with the density hypothesis (Unkelbach et al., 2008; Koch et al., 2016) which predicts a greater internal similarity of positive information. Accordingly, two additional positive features, being more similar, may have provided less positive evidence that would prompt individuals to alter their appraisal of an unfavourable candidate compared to two more differentiated negative features that carried sufficient information to diminish the evaluation of a favourable candidate. Alternatively, the absence of an effect of additional positive features on similarity judgments can be attributed to a lesser differentiation between negative options compared to positive ones, which is the focus of our current research (Jablonska et al., 2022).

### 4.3 Limitations and further research

Our study was not devoid of limitations. Firstly, in our descriptions, we exclusively utilised considerably positive and negative depictions of political candidates, leading to a ceiling effect in the evaluations of negative candidates’ profiles. Introducing intermediate steps could provide an opportunity to observe whether the weight of negative features surpasses the ratio-difference principle, akin to what we observed in the assessments of considerably positive candidates.

Secondly, employing more nuanced descriptions (i.e., with a broader range of proportions of negative and positive features) would enable us to examine if there exists any focusing effect concerning the target and the referent of evaluations. If we hypothesise that participants are less attuned to a larger number of features, it would be worthwhile to investigate whether there is any disparity in the sensitivity to the number of features of the target or the referent of similarity judgement. In essence, this would facilitate an exploration into whether the sensitivity parameters from Equation 2 differ for the common and distinctive features, even if they share the same valence.

Thirdly, we conducted only one study in which we investigated the relationship between objective and subjective similarity estimates and the effect of additional positive and negative features on similarity judgements. Although rather scarce on its own, the paper is a part of our string of publications on various instances of positive-negative asymmetry in similarity judgements (Falkowski et al., 2018, 2021; Falkowski and Jabłońska, 2018; Jablonska et al., 2022) and as such provides good evidence for the observed effects. Still, the comparison of normative predictions of the ratio model and subjective similarity estimates should be further verified on objects other than political candidates.

On the one hand, it is interesting whether the observed effects would hold for other, perhaps less emotion-laden objects. Politician evaluations are often driven by emotions, as indicated by previous studies (e.g., Johnston et al., 2015; Holloway and Wiener, 2023). Additionally, in our earlier research we found the affective evaluation to be a mediator between similarity judgements and voting intention (Falkowski and Jabłońska, 2018). Perhaps, the observed negativity effect is restricted to emotion-laden stimuli (such as politicians and social stimuli) which could explain the lack of this asymmetry in earlier discussed research on cities (Falkowski et al., 2021). Therefore, further studies should address this issue taking into account other objects such as other social stimuli or consumer products presented as in our studies as lists of features. Alternatively, in order to have a greater degree of control over the incremental changes in objective similarity, it is worth investigating objects presented numerically, e.g., with scales or rankings. Another intriguing avenue for research is to investigate whether the disparities between objective and subjective similarity judgments hold true for non-valenced stimuli, such as geometrical figures. Joining together the research on positive-negative asymmetry, similarity judgements and probability judgements mentioned in section “Discussion,” it is also interesting to investigate whether subjective probability judgements (of for instance a candidate being an effective leader or keeping their election promises) follow the subjective measures of similarity to an ideal and bad politician and, more interestingly, how this effect is moderated by candidate valence. Finally, researchers may be interested in studying in more detail differences between maximising and satisficing strategies and similarity and probability judgements.

## Data availability statement

The datasets presented in this study can be found in online repositories. The names of the repository/repositories and accession number(s) can be found below: https://osf.io/73p6t/?view_only=1f0a9d66bab142a5a44cb5ebeb3346aa.

## Ethics statement

The studies involving humans were approved by the Research Ethics Committee at SWPS University of Social Sciences and Humanities, Warsaw, Poland (approval number 12/2020). The studies were conducted in accordance with the local legislation and institutional requirements. Written informed consent from the patients/ participants or patients/participants’ legal guardian/next of kin was not required to participate in this study in accordance with the national legislation and the institutional requirements.

## Author contributions

MJ: Conceptualization, Data curation, Funding acquisition, Investigation, Methodology, Project administration, Writing – original draft. AF: Conceptualization, Supervision, Writing – review & editing. RM: Methodology, Supervision, Writing – review & editing.
