# Supplementary material for: Similarity judgements: the comparison of normative predictions and subjective evaluations – A study of the ratio model of similarity in social context
Source: Front Psychol. 2024 May 16;15:1335707. doi: 10.3389/fpsyg.2024.1335707 (PMC11139025; doi:10.3389/fpsyg.2024.1335707)
Supplement: Supplementary file 1 [file Presentation_1.pdf]

Supplementary Appendix: Additional information on candidate profiles and features used to describe politicians

A) *Candidate profiles used in the study. Features were selected based on previous research* (Falkowski & Jabłońska, 2018) *and the results of a pilot study (described later). In bold, marked features (either positive or negative) added to base profiles*

2+9-: The politician is disloyal, greedy, and lacks culture. They are despotic and quarrelsome. Their supporters praise them for caring for citizens and ensuring country security. Critics, however, reproach them for their populism and nepotism. They are considered a person who does not keep their promises and is lazy.

2+ 7-: The politician is disloyal, lazy, and greedy. They are despotic and have a quarrelsome character. Nevertheless, their supporters praise them for caring for citizens and ensuring country security. Critics, on the other hand, reproach them for their populism and nepotism.

4+9-: The politician is disloyal, greedy, and lacks culture. They are despotic and quarrelsome, but at the same time well-educated and committed. Their supporters praise them for caring for citizens and ensuring country security. Critics, however, reproach them for their populism and nepotism. They are considered a person who does not keep their promises and is lazy.

4+7-: The politician is disloyal, lazy, and greedy. They are despotic and quarrelsome, but at the same time well-educated and committed. Their supporters praise them for caring for citizens and ensuring country security. Critics, on the other hand, reproach them for their populism and nepotism.

7+4-: The politician is stable in their beliefs, consistent, and ambitious. They care for citizens and ensure country security. They have been criticized for their greediness and lack of culture. They are considered a person who does not keep their promises and is disloyal. Supporters emphasize their competence and the fact that they are a good speaker.

9+4-: The politician is stable in their beliefs, consistent, and ambitious. They care for citizens and ensure country security. They have been criticized for their greediness and lack of culture. They are considered a person who does not keep their promises and is disloyal. Supporters emphasize their competence and the fact that they are a good speaker. They are well-educated and committed.

7+2-: The politician is stable in their beliefs, consistent, and ambitious. They care for citizens and ensure country security. They have been criticized for their greediness and disloyalty. Supporters emphasize their competence and the fact that they are a good speaker.

9+2-: The politician is stable in their beliefs, consistent, and ambitious. They care for citizens and ensure country security. They have been criticized for their greediness and disloyalty. Supporters emphasize their competence and the fact that they are a good speaker. They are well-educated and committed.

B) The results of a pilot study in which we tested the combinations of particular features that were used in candidate profiles. In the study, we wanted to make sure that both base profiles and additional features (either positive or negative) differ in valence but not feature diagnosticity.

In the pilot study, sixteen participants were presented with a list of 17 positive and 17 negative features describing political candidates. The features were organised alphabetically. Respondents were asked to evaluate the extent of the positivity or negativity of each feature. Respondents provided their answers on 21-point Likert scales, with -10 as very negative and + 10 very positive with 0 in the middle. All features analysed in the pilot study are presented in Supplementary Table 1, along with their descriptive statistics. In order to select features for candidate profiles used the actual study, we chose features that were mutually exclusive and had similar affective loadings (when measured in absolute values). To make sure that basic seven positive features equaled basic seven negative features as well as those two additional favourable features equaled two additional unfavourable features, we ran two ANOVAs on the data from the pilot study. The results of conducted analyses showed that there were no differences between seven positive and seven negative features $F(1, 15) = 3.105$, $p = 0.098$, $\eta^2 = 0.172$ used to construct base profiles. Additionally, no differences between additional two positive and two negative features were found $F(1, 15) = 2.04$, $p = 0.173$, $\eta^2 = 0.120$.

Supplementary Table 1: Features analysed in the pilot study.

Positive features			Negative features			
	M	SD		M	SD	N
**ambitious**	5.44	2.73	**corrupted**	-8.25	2.70	16
**cares for citizens**	6.88	5.33	**despotic**	-4.50	5.50	16
**committed**	6.94	4.09	**disloyal**	-7.63	2.90	16
**competent**	7.00	3.88	**greedy**	-6.50	6.75	16
**consistent**	6.13	4.26	**incompetent**	-7.69	2.96	16
**educated**	6.06	3.70	**intolerant**	-2.56	4.77	16
**ensures country security**	8.50	2.68	**lacking culture**	-6.94	3.42	16
**experienced**	4.25	4.12	**lazy**	-7.88	3.30	16
**good public speaker**	4.94	2.65	**nepotistic**	-6.38	3.72	16
**sincere**	5.56	3.98	**not keeping election promises**	-7.81	2.83	16
**honest**	8.00	2.63	**partial**	-5.38	4.30	16
**impartial**	2.81	6.52	**populist**	-4.06	4.54	16
**just**	6.94	3.36	**quarrelsome**	-6.19	3.67	16
**keeping their word**	7.69	4.09	**radical**	-1.31	5.17	16
**loyal**	7.81	2.54	**stupid**	-7.81	3.10	16
**stable in their beliefs**	3.38	4.03	**thinking only about themselves**	-2.00	5.69	16
**truthful**	5.50	4.68	**uneducated**	-5.44	4.02	16
